# Role of systemic immune-inflammatory index and systemic inflammatory response index in predicting the diagnosis of necrotizing pneumonia in children

**DOI:** 10.1186/s12887-024-04818-8

**Published:** 2024-08-02

**Authors:** Rehab Elmeazawy, Dalia Ayoub, Lamia M. Morad, Ahmed Mohammed Farid EL-Moazen

**Affiliations:** 1 Department of Pediatrics, Faculty of Medicine, Tanta University, Tanta, Egypt; 2 Department of Pediatrics, Faculty of Medicine, Al-Azhar University, Assiut, Egypt

**Keywords:** Children, Necrotizing pneumonia, Systemic immune-inflammatory index, Systemic inflammation response index

## Abstract

**Background:**

Necrotizing pneumonia (NP) is a rare serious complication of community-acquired pneumonia (CAP) in children, which is characterized by a protracted course of the disease and a prolonged hospital stay. This study aimed to assess the role of systemic immune-inflammatory index and systemic inflammatory response index in predicting early lung necrotization in children with CAP.

**Methods:**

This study included all children hospitalized in Pediatric Pulmonology Unit, Tanta University, Egypt, with CAP between the ages of two months and 18 years. Systemic inflammatory indices, including the neutrophil/lymphocyte ratio (NLR), platelet/lymphocyte ratio (PLR), monocyte/lymphocyte ratio (MLR), systemic immune-inflammatory index (SII), and systemic inflammation response index (SIRI), were calculated on patients’ admission.

**Results:**

The study involved a total of 228 children, 42 patients had NP, 46 patients had parapneumonic effusion, and 140 patients had non-complicated CAP. Patients with NP were substantially younger (*p* = 0.002), stayed in the hospital longer (*p* < 0.001), had a longer duration of symptoms before hospital admission (*p* < 0.001), and had fever for a longer duration than those in the other groups (*p* < 0.001). Regarding the inflammatory ratios, patients with NP had significantly higher MLR, PLR, SII, and SIRI than those in the other groups (*p* = 0.020, *p* = 0.007, *p* = 0.001, *p* = 0.037, respectively). ROC curve analysis showed that the combined SII + SIRI + D-dimer showed the highest AUC with a good specificity in predicting the diagnosis of NP.

**Conclusions:**

SII, SIRI, and D-dimer may be beneficial biomarkers for predicting the occurrence of NP in children when performed on patients’ admission. In addition, it was found for the first time that combined SII + SIRI + D-dimer had a good sensitivity and specificity in the diagnosis of NP.

## Background

A rare form of community-acquired pneumonia (CAP) is necrotizing pneumonia (NP), which is marked by severe sickness, a protracted hospital stay, and a protracted course of the disease [1]. Streptococcus pneumoniae and Staphylococcus aureus are the most prevalent bacteria that cause NP, which exhibits lung necrosis, liquefaction, and loss of the typical pulmonary parenchymal architecture [2].

A growing number of NP cases in previously healthy children have been documented over the past 20 years [3]. The risk of complications from NP is also increased, including parapneumonic effusion, pneumothorax, pyopneumothorax, bronchopleural fistula, respiratory failure, septic shock, and death [4].

The most sensitive form of chest imaging for diagnosing NP is a CT scan, which can reveal multiple thin-walled cavities, decreased parenchymal enhancement, and loss of normal pulmonary architecture. Blood, pleural, sputum, and bronchoalveolar lavage (BAL) cultures can also be used for microbiological diagnosis [5].

Recent studies have shown that various combined ratios of complete blood count parameters, including NLR, PLR, MLR, SII, and SIRI, may serve as helpful diagnostic and prognostic indicators for various inflammatory conditions, including cancer [6], coronary artery disease [7], sepsis [8], and COVID-19 infection [9].

This study aimed to assess the role of systemic immune-inflammatory index and systemic inflammatory response index in predicting early lung necrotization in children with CAP to improve treatment and prognosis.

## Methods

This prospective cohort study included children admitted to the Pediatric Pulmonology Unit, Tanta University Hospital, Egypt, between September 2022 and August 2023 with a diagnosis of CAP. This study was performed in accordance with the principles of the Declaration of Helsinki. Approval was granted by the research ethics committee of the Faculty of Medicine, Tanta University (approval number 35,694/9/22). Written informed consent was obtained from the guardians of all patients.

All children aged between two months and 18 years who fulfilled the diagnostic criteria for CAP were included in this study. CAP was defined as the presence of signs and symptoms of pneumonia in a previously healthy child who acquired an infection outside the hospital and radiological findings of lung consolidation [10]. Children were diagnosed with parapneumonic effusion based on clinical examination and radiographic findings, whereas NP was diagnosed clinically based on the presence of progressive pneumonia in previously healthy children despite appropriate antibiotic therapy with a protracted course plus CT chest scans that revealed multiple necrotic foci in the consolidated area [11].

Children with the following criteria were excluded from the study: (1) Other causes of lung cavitation in radiological findings, such as solitary lung cavitation with defined rim of enhancement (lung abscess), congenital lung abnormalities, septic pulmonary embolism, or traumatic pseudocyst. (2) Chronic lung disorders, malignancy, underlying immunodeficiency, or neuromuscular illnesses. (3) Hospital-acquired pneumonia.

Patients were divided into three groups: Group 1 included patients with NP, Group 2 included patients with parapneumonic effusion (PPE), and Group 3 included patients with non-complicated pneumonia (CAP).

Demographic information was collected, including sex, age, characteristics of the initial clinical manifestations, length of hospitalization, duration of fever, occurrence of complications, and requirement for surgical intervention.

The duration of fever, the length of hospitalization, improvement in inflammatory markers, and the continuation of the same empirical antibiotics were objective measures to assess the recovery and the effectiveness of utilized antibiotics.

Laboratory tests, including complete blood count (CBC), were performed using two ml of venous blood in 20 μL EDTA solution using a Coulter® LH 700 series hematology analyzer to assess leukocyte count (10^3^/ μL), platelet count (10^3^/ μL), neutrophil count (10^3^/ μL), monocyte count (10^3^/ μL), lymphocyte count (10^3^/ μL) and quantitative C-reactive protein (CRP) measured by latex agglutination test, which is based on the immune chemical reaction between CRP and antibodies against CRP bound to latex particles. Positive results were obtained at concentrations of > 6 mg/L. D-dimer and procalcitonin levels were measured.

### Systemic inflammatory indices

The following formulations of the systemic inflammatory indices were used: NLR = Neutrophil/Lymphocyte, MLR = Monocyte/Lymphocyte, PLR = Platelet/ Lymphocyte, SII = (Platelet × Neutrophil)/Lymphocyte and SIRI = (Neutrophil × Monocyte)/Lymphocyte.

### Statistical analysis

Microsoft Excel 2020 was used to gather data and insert them into a spreadsheet. The statistical analysis was performed using SPSS version 23. Quantitative data with a normal distribution are expressed as mean and standard deviation (m ± SD) and were compared using the ANOVA test, whereas categorical data are displayed as numbers and percentages and were compared using the Kruskal Wallis test. To assess the prognostic significance of systemic inflammatory ratios for NP, receiver operating characteristic (ROC) curves were created. The optimal cut-off point was determined according to the Youden index. Based on univariate analysis, variables demonstrating statistical significance underwent multivariate logistic regression analysis to identify independent risk factors for NP. The exclusion of platelet, MLR, and PLR variables from the multivariate logistic regression analysis was deemed necessary due to their strong correlations with SII and SIRI. The goodness-of-fit of the logistic regression models was assessed using the Hosmer-Lemeshow test. The level of significance was set at *P* < 0.05.

## Results

### Clinical characteristics

**Fig. 1:**
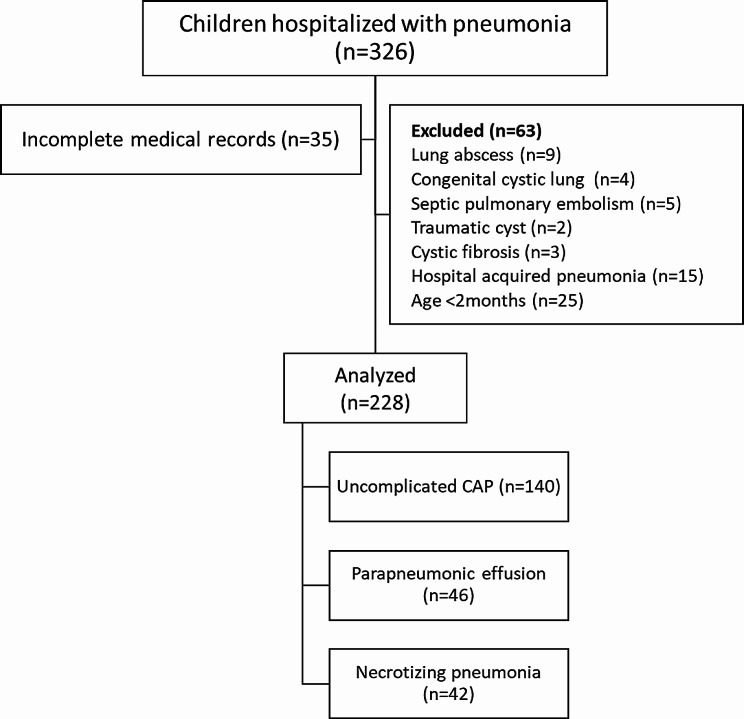
Flow chart of the study

A total of 326 patients were admitted to the hospital between September 2022 and August 2023 with a diagnosis of pneumonia (Fig. 1).

Of the 228 children included in the study, 42 (18.4%) NP, 46 (20.2%) had PPE, and 140 (61.4%) had noncomplicated CAP. The most frequent presenting symptom in each of the three groups was fever, whereas the most frequent symptoms in patients with NP were grunting (64.3%) and shortness of breath (57.1%), and the most frequent symptom in parapneumonic patients was gastrointestinal manifestations (vomiting and abdominal pain) (28.3%) (Table 1).

**Table 1:** Baseline characteristics of studied patients

	NP (*n* = 42)	PPE (*n* = 46)	CAP (*n* = 140)	*P* value
**Sex**				
▪ Male	21 (50.0%)	30 (65.2%)	84 (60.0%)	0.338
▪ Female	21 (50.0%)	16 (34.8%)	56 (40.0%)	
**Clinical manifestations**				
▪ Fever	42 (100.0%)	45 (97.8%)	138 (98.6%)	0.652
▪ Grunting	27 (64.3%)	19 (41.3%)	30 (21.4%)	< 0.001*
▪ Shortness of breath	24 (57.1%)	11 (23.9%)	18 (12.9%)	< 0.001*
▪ Chest pain	3 (7.1%)	5 (10.9%)	12 (8.6%)	0.823
▪ Cough	17 (40.5%)	21 (45.7%)	80 (57.1%)	0.194
▪ Hemoptysis	0 (0.0%)	0 (0.0%)	2 (1.4%)	0.531
▪ GIT symptoms	4 (9.5%)	13 (28.3%)	14 (10.0%)	0.013*
**Involved lung lobe**				< 0.001*
▪ Left lower	3 (7.1%)	7 (15.2%)	27 (38.6%)	
▪ Left upper	0 (0.0%)	1 (2.2%)	1 (1.4%)	
▪ Whole left side	11 (26.2%)	5 (10.9%)	2 (2.9%)	
▪ Right upper	1 (2.4%)	0 (0.0%)	9 (12.9%)	
▪ Right upper and middle	0 (0.0%)	0 (0.0%)	3 (4.3%)	
▪ Right middle and lower	0 (0.0%)	0 (0.0%)	2 (2.9%)	
▪ Right lower	6 (14.3%)	11 (23.9%)	16 (22.9%)	
▪ Whole right side	20 (47.6%	22 (47.8%)	5 (7.1%)	
▪ Bilateral lobar	1 (2.4%)	0 (0.0%)	5 (7.1%)	
**Ineffective antibiotics**	32 (76.2%)	30 (65.2%)	32 (22.9%)	< 0.001*
**Surgical intervention**	32 (76.2%)	35 (76.1%)	0 (0.0%)	< 0.001*
▪ Chest tube only	16 (38.1%)	23 (50.0%)		
▪ Chest tube + Lobectomy	1 (2.4%)	0 (0.0%)		
▪ Chest tube + Decortication	14 (33.3%)	5 (10.9%)		
▪ Chest tube + Cavernoplasty	1 (2.4%)	0 (0.0%)		
▪ Thoracocentesis	0 (0.0%)	4 (8.7%)		
▪ Chest tube e urokinase	0 (0.0%)	1 (2.2%)		
▪ Decortication	0 (0.0%)	2 (4.3%)		

*P value significant < 0.05, highly significant < 0.001, **NP**: necrotizing pneumonia, **PPE**: parapneumonic effusion, **CAP**: community-acquired pneumonia, **GIT**: gastrointestinal

The three groups differed significantly in terms of the involved lung lobes, ineffective antibiotics, and surgical drainage (*p* < 0.001) (Table 1). In the groups categorized as NP and PPE, the lung lobes that were most frequently affected were observed to be all lobes on the right side (47.6% and 47.8%, respectively). Subsequently, involvement of all lobes on the left side was documented in 26.2% of the NP group. Conversely, in the PPE group, the subsequent involved lobe was identified as the right lower lung lobe, affecting 23.9% of the patients. However, in the CAP group, the left lower lung lobe was found to be the prevailing site of affection (38.6%), followed by the right lower lobe in 22.9% of the patients (Table 1).

Pleural effusion was found in 76.2% of the patients with NP and the surgical intervention was performed in all of these patients, while in the PPE group, the surgical intervention was performed in 76.1% of the cases (Table 1). Pleural fluid analysis was performed in 22 (52.4%) of patients with NP and 31 (67.4%) of patients with PPE (Table 2). Pleural fluid culture was positive in 10 patients with NP and 11 patients with PPE.

**Table 2:** Laboratory findings among the studied groups

	NP (*n* = 42)	PPE (*n* = 46)	CAP (*n* = 140)	*P* value
**Age (years)** Median (IQR)	3.0 (1.6-4.0)	4.5 (2.9-7.0)	4.0 (2.5-6.0)	0.002*
**Duration of fever(days)** Median (IQR)	5.0 (3.0–7.0)	3.0 (2.0-6.3)	2.0 (1.0–3.0)	< 0.001*
**Duration of symptoms before admission (days)** Mean ± SD	7.81 ± 3.14	5.30 ± 1.87	2.74 ± 1.74	< 0.001*
**LOS (days)** Mean ± SD	17.31 ± 6.73	12.37 ± 4.78	7.95 ± 4.35	< 0.001*
**RR (breath/min)** Mean ± SD	56.83 ± 8.18	46.74 ± 9.32	48.13 ± 9.73	< 0.001*
**Hemoglobin (gm/dl)** Mean ± SD	9.55 ± 1.19	9.99 ± 1.72	9.87 ± 1.72	0.398
**HCT%** Mean ± SD	30.34 ± 4.22	31.17 ± 5.62	30.45 ± 4.92	0.677
**MCV (fl.)** Mean ± SD	74.40 ± 8.71	74.03 ± 8.42	72.78 ± 9.13	0.591
**MCH (pg/cell)** Mean ± SD	23.69 ± 3.35	23.79 ± 3.20	24.27 ± 7.04	0.822
**MCHC (g/cell)** Mean ± SD	31.77 ± 2.76	32.04 ± 2.30	32.23 ± 2.76	0.661
**RDW %** Mean ± SD	15.71 ± 1.95	14.96 ± 1.69	15.25 ± 2.53	0.267
**WBCs (10** ^**3**^ **/μL)** Median (IQR)	19.4 (14.35–25.16)	18.05 (13.38–24.88)	16.65 (12.28–23.1)	0.114
**Neutrophils (10** ^**3**^ **/μL)** Median (IQR)	15.5 (9.48-19.0)	13.7 (9.35–18.48)	11.9 (6.48–16.9)	0.063
**Lymphocytes (10** ^**3**^ **/μL)** Median (IQR)	3.6 (2.78–4.30)	3.9 (3.10–4.93)	4.2 (2.9–5.55)	0.457
**Monocytes (10** ^**3**^ **/μL)** Median (IQR)	0.9 (0.7–1.13)	0.8 (0.5–1.23)	0.7 (0.48-1.0)	0.085
**Platelets (10** ^**3**^ **/μL)** Median (IQR)	465.0 (363.3-556.5)	400.5 (309.5–553.0)	308.0 (256.8-442.3)	< 0.001*
**CRP (mg/l)** Median (IQR)	106.0 (82.0-155.0)	96.0 (63.8–167.0)	96.0 (55.3-189.3)	0.869
**NLR ratio** Median (IQR)	4.02 (2.70–6.10)	3.64 (2.69–4.89)	3.40 (1.55–5.32)	0.214
**MLR ratio** Median (IQR)	0.26 (0.18–0.32)	0.21 (0.14–0.30)	0.16 (0.10–0.31)	0.020*
**PLR ratio** Median (IQR)	118.09 (87.72-197.14)	100.27 (78.00-146.45)	86.77 (52.11-128.21)	0.007*
**SII** Median (IQR)	1702.4 (1257.9-2729.5)	1692.3(820.1-2322.4)	1043.4 (412.6-1888.6)	0.001*
**SIRI** Median (IQR)	3.28 (2.03–5.76)	2.40 (1.36–5.11)	2.23 (0.95–4.68)	0.037*
**Procalcitonin (ng/ml)** Median (IQR)	4.5 (1.1–7.5)	1.72 (0.75–4.79)	0.77 (0.58–1.13)	0.098
**D-dimer (μg/ml)** Median (IQR)	3.6 (2.1–5.9)	2.3 (1.5–4.4)	1.1 (0.4–2.3)	< 0.001*
**Pleural fluid analysis**				
**Glucose (mg/dl)** Median (IQR)	20.0 (10.0–33.0)	35.0 (11.0–68.0)		0.109
**Protein** (gm/dl) Median (IQR)	4.6 (4.3–5.48)	5.0 (4.0-5.5)		0.993
**TLC** (mm^3^) Median (IQR)	16,600 (7350–49,775)	12,800 (2900–63,500)		0.607
**Neutrophil** % Median (IQR)	87.5 (77.3–92.0)	80.0 (70.0–88.0)		0.087
**Lymphocyte**% Median (IQR)	12.5 (8.0-22.8)	20.0 (10.0–30.0)		0.141
**LDH** (U/L) Median (IQR)	5800 (1192-13495.5)	5925 (968-13157)		0.990
**ADA** (U/L) Median (IQR)	138.5 (68.1–165.0)	112.0 (30.3-161.3)		0.527

*P value significant < 0.05, highly significant < 0.001, **NP**: necrotizing pneumonia, **PPE**: parapneumonic effusion, **CAP**: community-acquired pneumonia, **LOS**: length of hospital stay, **RR**: Respiratory Rate, **HCT**; Hematocrit, **MCV**: Mean Corpuscular Volume, **MCH**: Mean Corpuscular Hemoglobin, **MCHC**: Mean Corpuscular Hemoglobin Concentration, **RDW**: Red Cell Distribution Width, **WBCs**: White Blood Cells, **CRP**: C-Reactive Protein, **NLR**: neutrophil/lymphocyte ratio, **MLR**: monocyte/lymphocyte ratio, **PLR**: platelet/ lymphocyte ratio, **SII**: systemic immune-inflammatory index, **SIRI**: systemic inflammatory response index, **TLC**: total leukocytic count, **LDH**: lactate dehydrogenase, **ADA**: adenosine deaminase

In the NP group, Methicillin-Sensitive Staphylococcus Aureus (MSSA) was the most common causative pathogen (*n* = 3), followed by Methicillin-Resistant Staphylococcus Aureus (MRSA) (*n* = 2), Pseudomonas aeruginosa (*n* = 2), Streptococcus pneumoniae (*n* = 1), mixed MSSA and Pseudomonas aeruginosa (*n* = 1), and Acinetobacter baumannii (*n* = 1). In the PPE group, MSSA was the most frequent organism (*n* = 6), followed by Streptococcus pneumonia (*n* = 3), MRSA (*n* = 1), and Klebsiella (*n* = 1).

### Laboratory findings

On admission, patients with NP had an increased platelet count (*p* < 0.001) compared with the other groups. Regarding the inflammatory ratios, patients with NP had significantly higher MLR, PLR, SII, SIRI, and D-dimer levels than those in the other groups. It is interesting to note that neither the NLR ratio nor the results of other laboratory tests differed significantly across the groups (Table 2).

### Treatment and outcome

The group of antibiotics that exhibited the highest level of responsiveness in the CAP group were Cephalosporin and Ampicillin sulbactam (35.7%), followed by Cephalosporin and Clindamycin (21.4%), while in PPE group, combined Carbapenem and Linezolid (28.3%) was the most effective antibiotics followed by Cephalosporin and Clindamycin (13.0%). In contrast, Linezolid and Clindamycin (47.6%) were the most effective antibiotics, followed by Carbapenem and Linezolid (19.0%).

Patients with NP were substantially younger, increased respiratory rate, stayed in the hospital longer, had a longer duration of symptoms before hospital admission, and had fever for a longer duration than those in the other groups (Table 2).

### Predictors for the diagnosis of NP

**Fig. 2:**
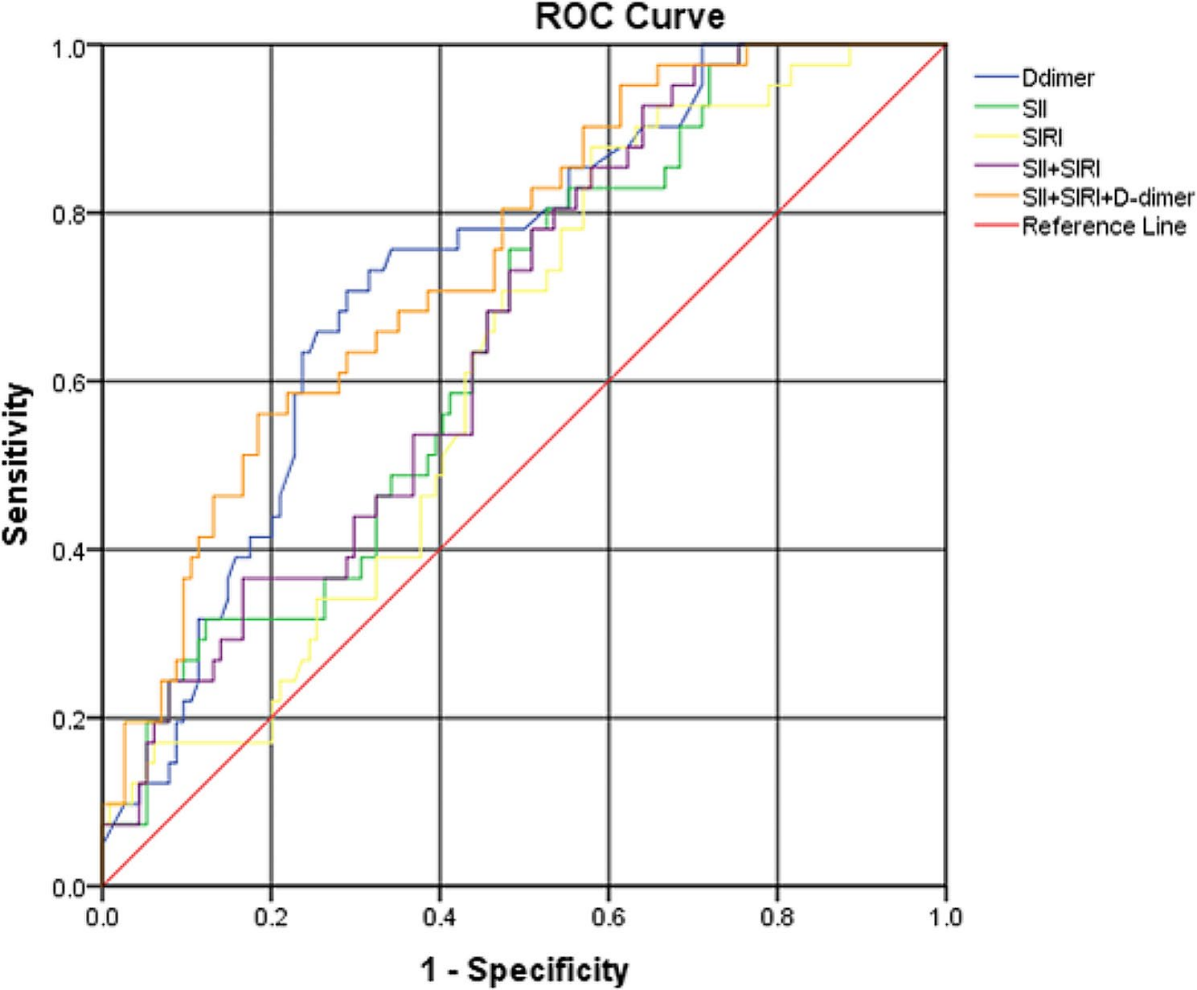
ROC curve analysis of SII, SIRI SII + SIRI, D-dimer, and SII + SIRI + D-dimer for the prediction of the diagnosis of NP

The area under the ROC curve for SII, SIRI, SII + SIRI, D-dimer, and SII + SIRI + D-dimer values on admission against patient outcome were 0.652 (95% CI 0.561–0.743), 0.620 (95% CI 0.528–0.713), 0.662 (95% CI 0.573–0.751), 0.730 (95% CI 0.646–0.814), and 0.745 (95% CI 0.661–0.828) respectively (Table 3) (Fig. 2). The combined SII + SIRI + D-dimer showed the highest AUC with a good specificity in predicting the diagnosis of NP.

**Table 3:** ROC curve analysis of predictors of NP

	Cutoff point	AUC	*P* value	Sensitivity	Specificity
SII	1124.06	0.652	< 0.001	80.5%	47.4%
SIRI	1.74	0.620	0.004	87.8%	42.1%
D-dimer	2.69	0.730	0.022	70.7%	71.1%
SII + SIRI	0.198	0.662	0.002	92.7%	36.0%
SII + SIRI + D-dimer	0.293	0.745	< 0.001	56.1%	81.6%

*P value significant < 0.05, highly significant < 0.001, **AUC**: area under the curve, **SII**: systemic immune-inflammatory index, **SIRI**: systemic inflammatory response index

Binary logistic regression revealed that factors that significantly increased the presence of NP even after adjustment were longer duration of symptoms before admission (OR: 1.810 (CI: 1.292–2.536), p: 0.001) and higher D-dimer (OR: 1.267 (CI: 1.057–1.518), p: 0.011). However, the presence of GIT manifestation (OR: 0.171 (CI: 0.032–0.909), p: 0.038) decreased the presence of NP (Table 4).

**Table 4:** Binary logistic regression analysis for factors determining necrotizing pneumonia among the study patients

Variables	OR (95% CI)	*P* value
Age (years)	0.855 (0.622–1.176)	0.336
Duration of symptoms before admission	1.810 (1.292–2.536)	0.001*
Duration of fever	1.171 (0.912–1.502)	0.215
Length of hospital say	1.049 (0.933–1.179)	0.427
Respiratory rate	1.082 (0.978–1.198)	0.126
Grunting	3.647 (0.491–27.085)	0.206
Shortness of breath	4.226 (0.585–30.552)	0.153
GIT manifestations	0.171 (0.032–0.909)	0.038*
SII	1.000 (1.000-1.001)	0.172
SIRI	1.019 (0.886–1.174)	0.789
D-Dimer	1.267 (1.057–1.518)	0.011*

OR: Odds ratio, CI: Confidence interval, *: Statistically significant at *p* ≤ 0.05, **GIT**: gastrointestinal tract, **SII**: systemic immune-inflammatory index, **SIRI**: systemic inflammatory response index

## Discussion

This study included 42 patients with NP who presented over the course of a year at a single institution. Because NP stimulates the inflammatory system, we investigated the effect of combined WBCs inflammatory ratios on the prediction of NP in children. MLR, PLR, SII, and SIRI were significantly higher in the NP group than in the other two groups, although WBCs and their differential cells were not statistically different across the three groups.

However, to the best of our knowledge, this is the first study to use SII, and SIRI as predictors of the diagnosis of NP, and no available data are available for comparison. These ratios have been used as prognostic markers in different fields, such as malignancy [12], ischemic stroke [13], systemic inflammatory disorders [14], and severe infections [15–17].

The diagnosis of NP as a unique entity of complicated pneumonia has previously been studied in a few small case series, with the largest research encompassing 282 patients over an 8-year period [18].

The median age of the patients with NP was 3 years, which is consistent with earlier research [19–21]. Despite the rapid course of NP and severe lung damage, both NP and PPE patients required surgical intervention at almost the same rate (76.2% and 76.1%, respectively) primarily involving pleural surgery. In contrast, lobectomy and cavernoplasty were performed in two patients with NP.

In contrast, Luo et al. observed that the NP group required much more surgical intervention, including lobectomy and pleural decortication, than the non-NP group, which may be explained by the fact that both groups of children were older when they began their studies, with a mean age of six years [22].

Although the precise pathophysiological process of NP remains unknown, it is distinguished by lung parenchymal necrosis, which is believed to be caused by the direct influence of bacterial toxins that produce inflammatory mediators and ultimately lead to tissue damage [23]. According to a different hypothesis, infections cause vasculitis, which stimulates the coagulation system and causes the development of microthrombi that obstruct intrapulmonary vessels, resulting in lung gangrene [24]. Both mechanisms lead to lung liquefaction and formation of multiple thin-walled cavities.

In light of this, the NP group in our study displayed considerably greater D-dimer levels than the PPE and CAP groups, which is consistent with prior results [22, 25].

An increased platelet count may play a vital role in the immune regulation process, as they participate in inflammatory processes such as coagulation, fibrinolysis, and tissue regeneration, interact with engulfed organisms, and release inflammatory cytokines [26].

In our study, NP patients had a significant increase in platelet count compared with the other groups, which is consistent with Seo et al., who reported a significantly higher platelet count in the NP group than in the non-NP group [27]. However, this result is in contrast to the findings reported by Luo et al., who found no significant difference in platelet counts between necrotizing and non-necrotizing groups [22]. This may be explained by the group selection of only mycoplasma NP patients, which may not have an effect on platelet count.

Prediction of NP is important for early diagnosis and proper management to avoid the occurrence of serious complications, such as massive tissue destruction, pneumonectomy, or even death. Thus, new indicators for the prediction of NP development should be identified along with classical diagnosis. SII, SIRI, D-dimer, and SII + SIRI + D-dimer appear to be optimistic tools for this purpose, as they are affordable, useful, and yield results quickly. Furthermore, it was discovered that they were more effective at identifying NP than traditional tests such as leukocyte counts and CRP.

Our study had some limitations, including a relatively small number of patients with NP, and it was a single-center study that only yielded suggestive results; therefore, future multicenter studies with larger sample sizes are necessary to verify the accuracy of the conclusions of this study.

## Conclusion

The use of systemic inflammatory indices (SII, SIRI, and D-dimer) for early NP prediction is crucial for favorable outcomes owing to the severity of NP and the high burden of hospitalization, particularly in nations with limited resources.

## Data Availability

The datasets used and/or analyzed during the current study are available from the corresponding author upon reasonable request.

## Abbreviations

CAP: Community-acquired pneumonia
CRP: C-reactive protein
MLR: Monocyte/lymphocyte ratio
MRSA: Methicillin-Resistant Staphylococcus Aureus
MSSA: Methicillin-Sensitive Staphylococcus Aureus
NLR: Neutrophil/lymphocyte ratio
NP: Necrotizing pneumonia
PLR: Platelet/lymphocyte ratio
PPE: Parapneumonic effusion
ROC: Receiver operating characteristic
SII: Systemic immune-inflammatory index
SIRI: Systemic inflammation response index
